# A new clitocyboid genus *Spodocybe* and a new subfamily Cuphophylloideae in the family Hygrophoraceae (Agaricales)

**DOI:** 10.3897/mycokeys.79.66302

**Published:** 2021-04-26

**Authors:** Zheng-Mi He, Zhu L. Yang

**Affiliations:** 1 CAS Key Laboratory for Plant Diversity and Biogeography of East Asia, Kunming Institute of Botany, Chinese Academy of Sciences, Kunming, 650201, China Kunming Institute of Botany, Chinese Academy of Sciences Kunming China; 2 Yunnan Key Laboratory for Fungal Diversity and Green Development, Kunming Institute of Botany, Chinese Academy of Sciences, Kunming, 650201, China Kunming Institute of Botany, Chinese Academy of Sciences Kunming China

**Keywords:** *
Ampulloclitocybe
*, *
Cantharocybe
*, *
Cuphophyllus
*, morphological characters, phylogenetic analysis, taxonomy

## Abstract

Phylogenetically, the genera *Cuphophyllus*, *Ampulloclitocybe* and *Cantharocybe* are treated as basal in the family Hygrophoraceae, despite weak support. However, the exact phylogenetic positions of the three genera have remained unresolved, and taxa related to these genera are poorly known. In this study, a new clitocyboid genus *Spodocybe* was proposed based on multigenic phylogenetic inference datasets and morphological evidence. The analyses of ITS as well as two combined datasets ITS-nrLSU-*rpb2* and ITS-nrLSU-*rpb1*-*rpb2*-*tef1-α*-*atp6* supported that (1) *Spodocybe* formed a well-supported monophyletic clade; and (2) sisters *Spodocybe* and *Ampulloclitocybe*, along with *Cantharocybe* and *Cuphophyllus* also formed a monophyletic lineage, as sister to the rest of the Hygrophoraceae. Meanwhile, two new species, namely *S. rugosiceps* and *S. bispora*, from southwestern China, were documented and illustrated. These results support the new proposed genus *Spodocybe*, and that *Spodocybe*, *Ampulloclitocybe*, *Cantharocybe* and *Cuphophyllus* should be retained in the Hygrophoraceae as a new subfamily Cuphophylloideae.

## Introduction

The widespread genus *Clitocybe* (Fr.) Staude currently encompasses large numbers of species with clitocyboid habit, sharing the features of saprophytic nutrition, funnel-shaped pileus, decurrent lamellae, a usually white, cream or pale colored spore-deposit and smooth and inamyloid spores (Singer 1986; Breitenbach and Kraenzlin 1991; Læssøe and Petersen 2019). As a consequence of the poor, broad and unrepresentative morphological characteristics, the genus appeared heterogeneous and was subsequently proven to be polyphyletic based on the phylogenetic analysis (Moncalvo et al. 2002; Harmaja 2003).

Based on phylogenetic analyses over the past 20 years, (i) many new genera within the Tricholomatoid clade were proposed to accommodate previous *Clitocybe* species deviating from the core Clitocybeae clade (Matheny et al. 2006), such as *Cleistocybe* Ammirati, A.D. Parker & Matheny (Ammirati et al. 2007), *Trichocybe* Vizzini (Vizzini et al. 2010), *Atractosporocybe* P. Alvarado, G. Moreno & Vizzini, *Leucocybe* Vizzini, P. Alvarado, G. Moreno & Consiglio and *Rhizocybe* Vizzini, G. Moreno, P. Alvarado & Consiglio (Alvarado et al. 2015); (ii) Several clitocyboid groups were reconfirmed as independent genera, for instance, *Singerocybe* Harmaja (Qin et al. 2014) and *Infundibulicybe* Harmaja (Binder et al. 2010); and (iii) some others were even transferred to the Hygrophoroid clade (Binder et al. 2010), such as *Ampulloclitocybe* Redhead, Lutzoni, Moncalvo & Vilgalys (Redhead et al. 2002) and *Cantharocybe* H.E. Bigelow & A.H. Sm. (Hosen et al. 2016). However, many clitocyboid taxa remain to be reclassified.

The molecular phylogenetic relationships among members of the Hygrophoraceae Lotsy were well studied by Lodge et al. (2014). In their work, the family was divided into subfamily Hygrophoroideae E. Larss., Lodge, Vizzini, Norvell & S.A. Redhead, Hygrocyboideae Padamsee & Lodge, Lichenomphalioideae Lücking & Redhead and Cuphophylloid grade. Meanwhile, the Cuphophylloid grade was retained in the Hygrophoraceae as the base comprising the genera *Cuphophyllus* (Donk) Bon, *Ampulloclitocybe* and *Cantharocybe*, despite weak phylogenetic support (Matheny et al. 2006; Binder et al. 2010; Lodge et al. 2014). Consequently, the taxonomic problem of the three genera on whether to be included or excluded in the Hygrophoraceae has remained unresolved.

Recently, some collections were shown to be closely related to *Clitocybe trulliformis* (Fr.) P. Karst. based on ITS-BLAST searches while at the same time they were surprisingly related to taxa of the genus *Cuphophyllus* based on nrLSU-BLAST searches. As far as we know, *C. trulliformis* and allied species were lacking taxonomic revision, especially regarding their molecular phylogenetic status. Furthermore, the phylogenetic delimitation of the Hygrophoraceae was ambiguous due to the uncertain positions of *Cuphophyllus*, *Ampulloclitocybe* and *Cantharocybe*. Hence, the aims of this study were (a) to propose and describe a new genus of the Hygrophoraceae for species related to *C. trulliformis* based on morphological and molecular analyses and (b) to reconstruct the phylogeny of the Hygrophoraceae for determining the exact phylogenetic placements of *Cuphophyllus*, *Ampulloclitocybe* and *Cantharocybe* with multi-gene data.

## Materials and methods

### Specimens

Twenty-three specimens of species similar to *C. trulliformis* and related species were collected from southwestern and northeastern China and western Germany, during 2007 to 2020. The fresh fruitbodies were dried using heat or silica gel. Voucher specimens were deposited in the Herbarium of Kunming Institute of Botany, Chinese Academy of Sciences (KUN-HKAS). Detail information of these specimens is given in Table 1.

**Table 1.** Specimens used in phylogenetic analysis and their GenBank accession numbers. The newly generated sequences are shown in bold.

Species	Voucher	Locality	GenBank accession number					
			ITS	nrLSU	*rpb2*	*rpb1*	*tef1-α*	*atp6*
*Acantholichen pannarioides*	MDF352	Costa Rica	KT429795	KT429807	KT429817			
*Acantholichen campestris*	DIC595b	Brazil	KT429798	KT429810	KT429818			
*Acantholichen galapagoensis*	MDF058	Ecuador	KT429785	KT429800	KT429812			
*Ampulloclitocybe clavipes*	KUN-HKAS 54426	China: Jilin	**MW616462**	**MW600481**	**MW656471**	**MW656467**	**MW656461**	**MW656478**
	AFTOL-ID 542		AY789080	AY639881	AY780937	AY788848	AY881022	
	DJL06TN40	USA	FJ596912	KF381542	KF407938			
*Arrhenia auriscalpium*	TUB 011588			DQ071732				
*Arrhenia acerosa*	Lueck2	Germany	KP965766	KP965784				
*Cantharellula umbonata*	CBS 398.79	France	MH861222	MH872990				
*Cantharocybe gruberi*	AFTOL-ID 1017	USA	DQ200927	DQ234540	DQ385879	DQ435808	DQ059045	
	AH24539	Spain	JN006422	JN006420				
*Cantharocybe brunneovelutina*	DJL-BZ-1883	Belize	NR160458	NG068731				
*Cantharocybe virosa*	TENN63483	India	KX452405	JX101471				
	Iqbal-568	Bangladesh	KX452403	KF303143				
*Chromosera cyanophylla*	AFTOL-ID 1684	USA	DQ486688	DQ457655	KF381509			
*Chromosera ambigua*	GE18008-1	France	MK645573	MK645587	MK645593			
*Chromosera lilacina*	GE18035	Canada	MK645577	MK645591	MK645597			
*Chromosera xanthochroa*	GE18033	Canada	MK645576	MK645590	MK645596			
*Chrysomphalina chrysophylla*	AFTOL-ID 1523	USA	DQ192180	DQ457656				
*Chrysomphalina grossula*	OSC 113683		EU644704	EU652373				
Clitocybe aff. costata	DJL06TN80	USA	FJ596913					
*Clitocybe herbarum*	G0171	Hungary		MK277719				
*Clitocybe trulliformis*	14562	Italy	JF907809					
	4804	Russia	MH930178					
Clitocybe cf. trulliformis	G0460	Hungary		MK277728				
*Clitocybe* sp.	NAMA 2015-206	USA	MH910535					
*Clitocybe* sp.	NAMA 2015-318	USA	MH910563					
*Clitocybe* sp.	Mushroom Observer 302917	USA	MK607556					
*Cora pavonia*	DIC215	Ecuador	KF443238	KF443261	KF443275			
*Cora aspera*	DIC110	Bolivia	KF443230	KF443257	KF443267			
*Cora reticulifera*	DIC119	Ecuador	KF443239	KF443262	KF443269			
*Cora squamiformis*	DIC146	Bolivia	KF443240	KF443263	KF443273			
*Corella brasiliensis*	MDF017	Bolivia	KF443229	KF443255	KF443276			
*Corella* aff. *Melvinii*	MDF200	Brazil	KJ780569	KY861725				
*Cuphophyllus pratensis*	Lueck7	Germany	KP965771	KP965789				
	DJL-Scot-8	UK	KF291057	KF291058				
*Cuphophyllus aurantius*	CFMR PR-6601	Puerto Rico	KF291099	KF291100	KF291102			
Cuphophyllus aff. pratensis	AFTOL-ID 1682	USA	DQ486683	DQ457650		DQ435804		
*Cuphophyllus* sp.	KUN-HKAS 105671	China: Tibet	**MW762875**	**MW763000**	**MW789179**	**MW789163**		
*Cyphellostereum galapagoense*	CDS 41163	Ecuador	NR158415	NG068806				
*Cyphellostereum imperfectum*	DIC115	Guatemala	KF443218	KF443243	KF443277			
*Dictyonema interruptum*	Ertz 10475	Portugal		EU825967	KF443282			
*Dictyonema schenckianum*	DIC113	Brazil	KF443225	KF443251	KF443285			
*Eonema pyriforme*	G1063	Poland		MK278075				
*Gliophorus psittacinus*	CFMR DEN-25	Denmark	KF291075	KF291076	KF291078			
*Gliophorus graminicolor*	TJB-10048 (CORT)	Australia	KF381520	KF381545	KF407936			
Gliophorus aff. laetus	CFMR PR-5408	Puerto Rico	KF291069	KF291070				
*Gloioxanthomyces nitidus*	GDGM41710	China: Jilin	MG712283	MG712282	MG711911			
*Haasiella splendidissima*	Herb. Roux n. 4044	France	JN944400	JN944401				
	Herb. Roux n. 3666	Moldova	JN944398	JN944399				
*Haasiella venustissima*	A. Gminder 971488	Italy	KF291092	KF291093				
	E. C. 08191	Italy	JN944393	JN944394				
*Humidicutis marginata*	JM96/33			AF042580				
*Humidicutis auratocephalus*	AFTOL-ID 1727	USA	DQ490624	DQ457672	DQ472720	DQ447906		
*Humidicutis dictiocephala*	QCAM6000	Ecuador	KY689661	KY780120				
*Humidicutis* sp.	CFMR BZ-3923	Belize	KF291110	KF291111				
*Hygroaster nodulisporus*	AFTOL-ID 2020	USA		EF561625				
*Hygroaster albellus*	AFTOL-ID 1997	Puerto Rico	KF381521	EF551314				
*Hygrocybe conica*	FO 46714			DQ071739				
Hygrocybe cf. acutoconica	CFMR NC-256	USA	KF291117	KF291118	KF291120			
*Hygrocybe coccinea*	AFTOL-ID 1715	USA	DQ490629	DQ457676	DQ472723	DQ447910	GU187705	
Hygrocybe aff. conica	AFTOL-ID 729		AY854074	AY684167	AY803747			
*Hygrophorus eburneus*	US97/138	Germany		AF430279				
	GDGM70059	USA	MT093608					
*Hygrophorus chrysodon*	KUN-HKAS 82501	China: Tibet	**MW616463**	**MW600482**	**MW656472**		**MW656462**	**MW656479**
	KUN-HKAS 112569	China: Tibet	**MW762876**	**MW763001**	**MW789180**	**MW789164**	**MW773440**	**MW789195**
*Hygrophorus flavodiscus*	KUN-HKAS 68013	China: Yunnan	**MW616464**	**MW600483**	**MW656473**	**MW656468**	**MW656463**	**MW656480**
	KUN-HKAS 55043	China: Yunnan	**MW616465**	**MW600484**	**MW656474**	**MW656469**	**MW656464**	**MW656481**
*Hygrophorus gliocyclus*	KUN-HKAS 79929	China: Tibet	**MW616466**	**MW600485**	**MW656475**		**MW656465**	**MW656482**
*Hygrophorus hypothejus*	KUN-HKAS 56550	Germany	**MW616467**	**MW600486**	**MW656476**	**MW656470**		**MW656483**
*Hygrophorus pudorinus*	AFTOL-ID 1723	USA	DQ490631	DQ457678	DQ472725	DQ447912	GU187710	
*Hygrophorus* sp. 1	KUN-HKAS 112566	China: Yunnan	**MW762877**	**MW763002**	**MW789181**	**MW789165**	**MW773441**	**MW789196**
*Hygrophorus* sp. 2	KUN-HKAS 87261	China: Jilin	**MW616468**	**MW600487**	**MW656477**		**MW656466**	**MW656484**
*Hygrophorus* sp. 3	KUN-HKAS 112567	China: Tibet	**MW762878**	**MW763003**	**MW789182**	**MW789166**	**MW773442**	**MW789197**
*Hygrophorus* sp. 4	KUN-HKAS 112568	China: Tibet	**MW762879**	**MW763004**	**MW789183**	**MW789167**	**MW773443**	**MW789198**
*Lichenomphalia hudsoniana*	GAL18249	USA	JQ065873	JQ065875				
*Lichenomphalia meridionalis*	S-270-FB1	Japan	LC428308	LC428307				
*Neohygrocybe ovina*	GWG H. ovina Rhosisaf (ABS)	UK	KF291233	KF291234	KF291236			
*Neohygrocybe griseonigra*	GDGM 44492	China	MG779451	MG786565				
*Neohygrocybe ingrata*	DJL05TN62 (TENN)	USA	KF381525	KF381558	KF381516			
*Neohygrocybe subovina*	GRSM 77065	USA	KF291140	KF291141				
*Spodocybe bispora*	KUN-HKAS 73310	China: Yunnan	**MW762880**	**MW763005**	**MW789184**	**MW789168**	**MW773444**	**MW789199**
	KUN-HKAS 73332	China: Yunnan	**MW762881**	**MW763006**	**MW789185**	**MW789169**	**MW773445**	**MW789200**
	KUN-HKAS 112564	China: Yunnan	**MW762882**	**MW763007**	**MW789186**	**MW789170**	**MW773446**	**MW789201**
*Spodocybe rugosiceps*	KUN-HKAS 112561	China: Yunnan	**MW762883**	**MW763008**	**MW789187**	**MW789171**	**MW773447**	**MW789202**
	KUN-HKAS 81981	China: Yunnan	**MW762884**	**MW763009**	**MW789188**	**MW789172**		**MW789203**
	KUN-HKAS 69830	China: Yunnan	**MW762885**	**MW763010**	**MW789189**	**MW789173**	**MW773448**	**MW789204**
*Spodocybe rugosiceps*	KUN-HKAS 71071	China: Yunnan	**MW762886**	**MW763011**	**MW789190**	**MW789174**	**MW773449**	**MW789205**
	KUN-HKAS 112562	China: Yunnan	**MW762887**	**MW763012**	**MW789191**	**MW789175**	**MW789159**	**MW789206**
	KUN-HKAS 112563	China: Yunnan	**MW762888**	**MW763013**	**MW789192**	**MW789176**	**MW789160**	**MW789207**
*Spodocybe* sp. 1	KUN-HKAS 112560	China: Jilin	**MW762889**	**MW763014**	**MW789193**	**MW789177**	**MW789161**	**MW789208**
*Spodocybe* sp. 2	KUN-HKAS 112565	China: Yunnan	**MW762890**	**MW763015**	**MW789194**	**MW789178**	**MW789162**	**MW789209**
*Porpolomopsis calyptriformis*	CFMR ENG-3	UK	KF291242	KF291243	KF291245			
Porpolomopsis aff. calyptriformis	DJL05TN80 (TENN)	USA	KF291246	KF291247	KF291249			
*Porpolomopsis lewelliniae*	TJB-10034 (CORT)	Thailand	KF291238	KF291239	KF291241			
*Pseudoarmillariella ectypoides*	AFTOL-ID 1557	USA	DQ192175	DQ154111	DQ474127	DQ516076	GU187733	
*Pseudoarmillariella bacillaris*	KUN-HKAS 76377	China	KC222315	KC222316				
*Sinohygrocybe tomentosipes*	GDGM 50075	China: Hunan	MG685873	MG696902	MG696906			
	GDGM 43351	China: Sichuan	MG685872	MG696901	MG696905			
*Amylocorticium cebennense*	CFMR HHB-2808	USA	GU187505	GU187561	GU187770	GU187439	GU187675	
*Aphroditeola olida*	DAOM 226047	Canada	KF381518	KF381541				
*Macrotyphula fistulosa*	IO. 14. 214	Spain	MT232352	KY224088		MT242317	MT242354	
*Macrotyphula juncea*	IO. 14. 177	Sweden	MT232353	MT232306	MT242337		MT242355	
*Macrotyphula phacorrhiza*	IO. 14. 167	Sweden	MT232364	MT232315	MT242348	MT242326	MT242367	
	IO.14. 200	France	MT232363	MT232314	MT242347		MT242366	
*Phyllotopsis nidulans*	IO. 14. 196	Spain	MT232308		MT242338	MT242319	MT242357	
*Phyllotopsis* sp.	AFTOL-ID 773		DQ404382	AY684161	AY786061	DQ447933	DQ059047	
*Pleurocybella porrigens*	UPS F-611822	Sweden	MT232355	MT232309	MT242339			
*Plicaturopsis crispa*	AFTOL-ID 1924	USA	DQ494686	DQ470820	GU187816			
*Pterulicium echo*	ZRL20151311		LT716065	KY418881	KY419026	KY418979	KY419076	
*Pterulicium gracilis*	IO. 14. 142	Sweden	MT232356	MT232310			MT242358	
*Sarcomyxa serotina*	AFTOL-ID 536	USA	DQ494695	AY691887	DQ859892	DQ447938	GU187754	
*Serpulomyces borealis*	CFMR L-8014	USA	GU187512	GU187570	GU187782		GU187686	
*Tricholomopsis decora*	AFTOL-ID 537		DQ404384	AY691888	DQ408112	DQ447943	DQ029195	
*Tricholomopsis osiliensis*	ZRL20151760		LT716068	KY418884	KY419029		KY419079	
*Typhula capitata*	IO. 15. 122	Spain	MT232357	MT232312	MT242341	MT242321	MT242360	
*Typhula incarnata*	IO. 14. 92	Sweden	MT232362	MT232313	MT242346	MT242325		
*Typhula micans*	IO. 14. 165	Sweden	MT232361	KY224102	MT242345	MT242324	MT242364	

### Morphological observation

Macroscopic characters of species were described based on the raw field record data and photographs. Colors used in description referred to Kornerup and Wanscher (1978). For the microscopic structure observation, tissue sections of dried specimens were mounted in 5% KOH solution or distilled water and structures of lamellar trama, pileipellis and stipitipellis, basidia and basidiospores were observed with a light microscopy. For the description of lamellar trama structure, seven types, including regular, subregular, divergent, pachypodial, bidirectional, tri-directional and interwoven, were used following Lodge et al. (2014). Besides, Melzer’s reagent was applied to test the amyloidity of the basidiospores. In the description of basidiospores, the abbreviation [n/m/p] represent that the measurements were made on n basidiospores from m basidiomes of p collections. The range notation (a)b–c(d) stands for the dimensions of basidiospores in which b–c contains a minimum of 90% of the measured values while a and d in the brackets stand for the extreme values. In addition, a Q value show the length/width ratio of basidiospores and a Qm value for average Q ± standard deviation. All microstructures were illustrated by hand drawing.

### DNA extraction, PCR and sequencing

Total genomic DNA was extracted using the Ezup Column Fungi Genomic DNA Purificaton Kit (Sangon Biotech, Shanghai, China) according to the manual. For the PCR amplification, (1) Primers ITS5 and ITS4 (White et al. 1990) were used for the internal transcribed spacer (ITS); (2) LROR and LR5 (Vilgalys and Hester 1990) for the nuclear ribosomal large subunit (nrLSU); (3) EF1-983F and EF1-1953R (Matheny et al. 2007), designed primers SPO-TEF1-F (5’-ATTGCYGGYGGTACYGGTGA-3’) and SPO-TEF1-R (5’-TCVAGDGATTTACCTGTHCGRC-3’) or another pair of designed primers HYG-TEF1-F (5’-CTTGCCTTYACTCTYGGYGTCC-3’) and HYG-TEF1-R (5’-GCGAACTTGCASGCAATGTG-3’) for the translation elongation factor 1-α (*tef1-α*); (4) RPB1-Af and RPB1-Cr (Matheny et al. 2002) or designed primers SPO-RPB1-F (5’-ACGAGGTTGYGTGGTGAAAT-3’) and SPO-RPB1-R (5’-GGAGGNGGDACHGGCATNA-3’) for the DNA-directed RNA polymerase II second largest subunit 1 (*rpb1*); (5) RPB2-6F and RPB2-7.1R (Matheny 2005) for the DNA-directed RNA polymerase II second largest subunit 2 (*rpb2*); and (6) ATP6-3 and ATP-6 (Kretzer and Bruns 1999) for ATP synthase subunit 6 (*atp6*).

The PCR mixtures contained 1× PCR buffer, 1.5mM MgCl_2_, 0.2mM dNTPs, each primer at 0.4 μM, 1.25U of *Taq* polymerase (Sangon Biotech, Shanghai, China), and 1 μL of DNA template in a total volume of 25 μL. Reactions were performed with the following program: initial denaturation at 94 °C for 5 min, 35 cycles at 94 °C for 30 s, 50 °C (*atp6*), 52 °C (nrLSU, *tef1-α*, *rpb1* and *rpb2*) or 54 °C (ITS) for 30 s, and 72 °C for 30 s (ITS and *atp6*), 50 s (nrLSU and *rpb2*) or 75 s (*tef1-α* and *rpb1*), and for terminal elongation, the reaction batches were incubated at 72 °C for 10 min. All PCR products were detected by 2% agarose gel electrophoresis and then sent to the Kunming branch of Tsingke Biological Technology Co., Ltd. (Beijing, China) for sequencing.

### Phylogenetic tree construction

Sequences used for phylogenetic analysis (presented in Table 1) were aligned by using MAFFT v7.471 (Katoh and Standley 2016) and then manually adjusted by using BIOEDIT v7.2.5 (Hall 1999). The intron regions of *tef1-α*, *rpb2* and *rpb1* were excluded except the conserved rpb1-intron2. Three datasets of ITS-nrLSU-*rpb2*, ITS-nrLSU-*rpb1*-*rpb2*-*tef1-α*-*atp6* and ITS (Suppl. materials 1, 2 and 3) were used to construct phylogenetic trees. The two multi-gene matrixes were generated by SEQUENCEMATRIX 1.7.8 (Vaidya et al. 2011). GTR + I + G was inferred as the best-fit model for the three matrixes selected according to the AIC in MRMODELTEST v2.4 (Nylander 2004). Maximum likelihood (ML) trees with 1000 bootstrap replicates and Bayesian inferences were generated with RAXML v8.0.20 (Stamatakis 2006) and MRBAYES v3.2.7 (Ronquist and Huelsenbeck 2003), respectively.

## Results

### Molecular phylogenetic analysis

As shown in Table 1, a total of 393 sequences (109 ITS, 110 nrLSU, 40 *tef1-α*, 38 *rpb1*, 74 *rpb2* and 22 *atp6*) from 118 samples were used in the phylogenetic analyses, 131 (23 ITS, 23 nrLSU, 20 *tef1-α*, 20 *rpb1*, 23 *rpb2* and 22 *atp6*) of which were newly generated in the present study.

**Figure 1.**
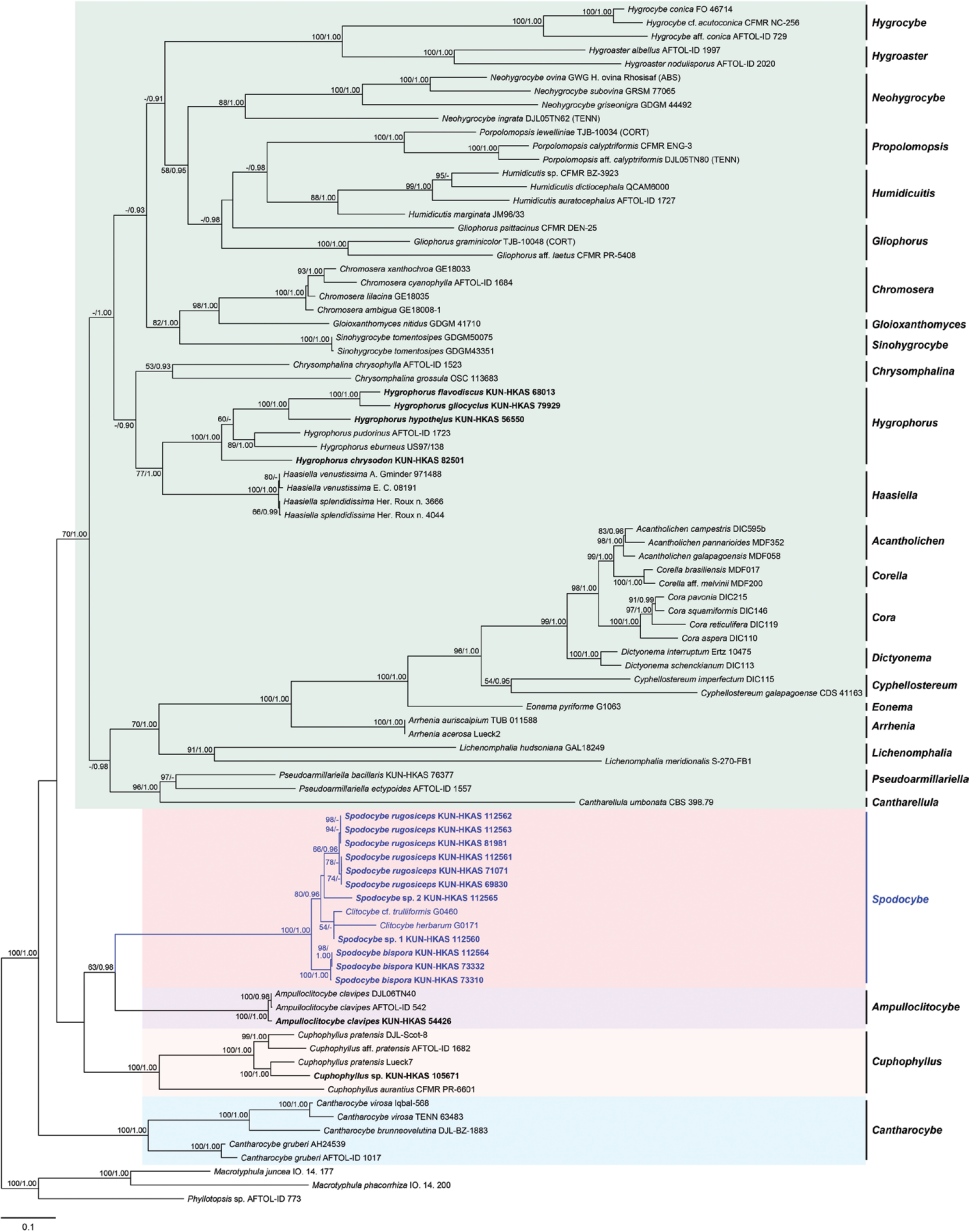
ML analysis of Hygrophoraceae combined ITS, nrLSU and *rpb2* sequence data, with *Macrotyphula juncea*, *Macrotyphula phacorrhiza* and *Phyllotopsis* sp. as outgroups. Bootstrap values (BP) ≥ 50% from ML analysis and Bayesian posterior probabilities (PP) ≥ 0.90 are shown at nodes. The newly generated sequences are shown in bold.

The combined dataset ITS-nrLSU-*rpb2* comprised 221 sequences from 88 samples with a total of 3135 positions. In the three-gene tree (Fig. 1), 11 specimens from four novel *Spodocybe* species collected in this study, C. cf. trulliformis and *C. herbarum* formed a strongly supported monophyletic clade (BP = 100%, PP = 1.0), as sister to *Ampulloclitocybe* (BP = 63%, PP = 0.98). The phylogenetic analysis showed that the new proposed genus *Spodocybe* should be placed within the Hygrophoraceae, although intergeneric branched orders among *Spodocybe*, *Ampulloclitocybe*, *Cantharocybe* and *Cuphophyllus* were unstable with low support values.

**Figure 2.**
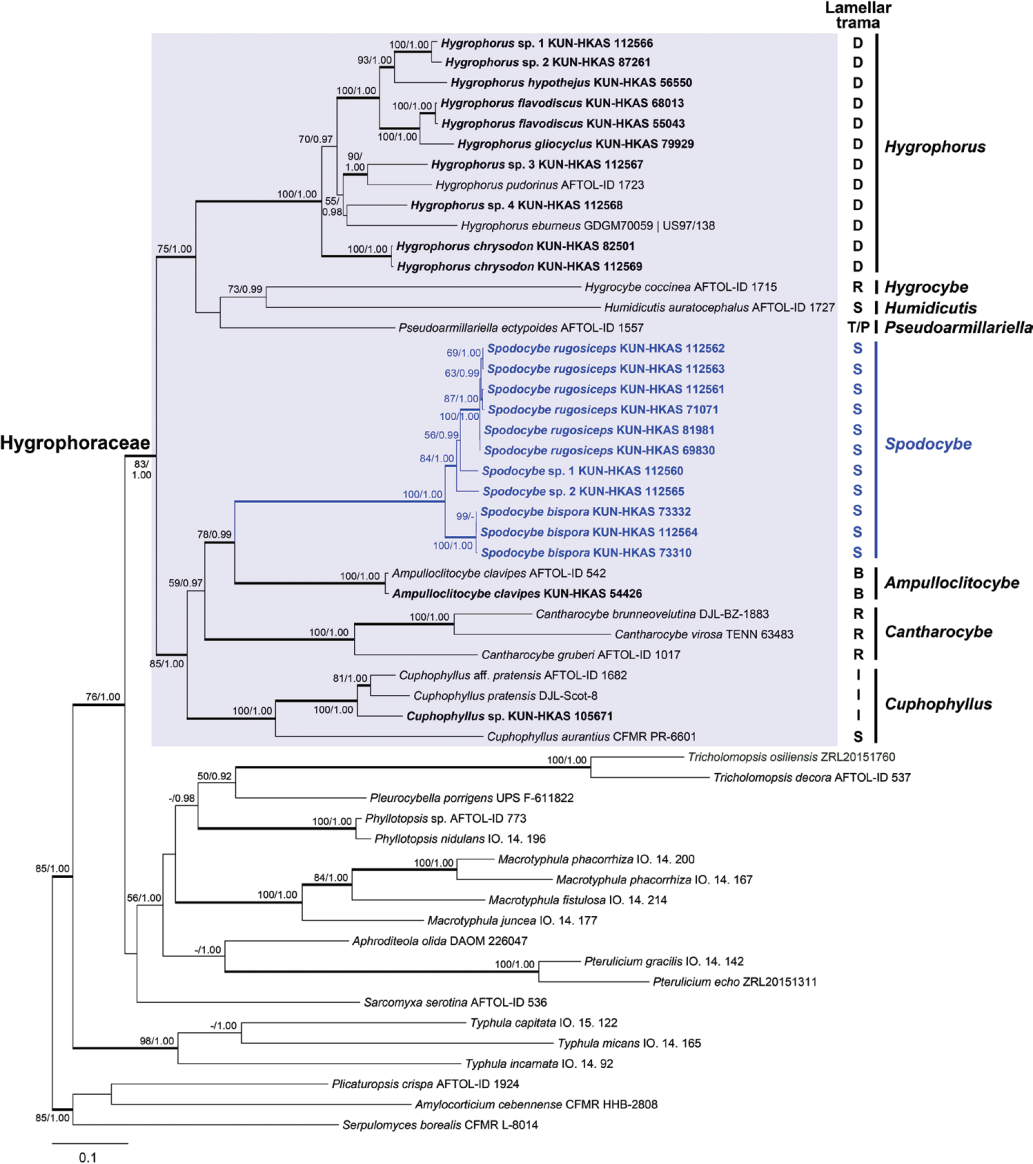
ML analysis of Hygrophoraceae combined ITS, nrLSU, *rpb1*, *rpb2*, *tef1-α* and *atp6* sequence data, with representatives of Amylocorticiaceae, Pterulaceae and the Hygrophoroid clade (*Aphroditeola*, *Macrotyphula*, *Phyllotopsis*, *Pleurocybella*, *Sarcomyxa*, *Tricholomopsis* and *Typhula*) as outgroups. Bootstrap values (BP) ≥ 50% from ML analysis and Bayesian posterior probabilities (PP) ≥ 0.90 are shown at nodes. Branches with BP ≥ 75% and PP ≥ 0.95 are bolded. The newly generated sequences are shown in bold. Lamellar trama type B for bidirectional, D for divergent, I for interwoven, P for pachypodial, R for regular, S for subregular, T for tri-directional. Lamellar trama types of specimens collected in this study were identified by ourselves and others referred to Lodge et al. (2014) and Hosen et al. (2016).

In order to accurately determine the position of *Spodocybe* in the family Hygrophoraceae and better clarify the phylogenetic relationships of *Spodocybe*, *Ampulloclitocybe*, *Cantharocybe* and *Cuphophyllus*, a further six-gene matrix ITS-nrLSU-*rpb1*-*rpb2*-*tef1-α*-*atp6* composed of 179 sequences from 54 samples with 5405 positions was used to rebuild the Hygrophoraceae tree. As revealed by the six-gene phylogenetic analysis (Fig. 2), the branch support level of the six-gene tree was obviously improved, compared with that of the previous three-gene tree. The monophyly of *Spodocybe* clade was strongly supported (BP = 100%, PP = 1.00), including *Spodocybe rugosiceps* (BP = 100%, PP = 1.00), *S. bispora* (BP = 100%, PP = 1.00) and two unnamed *Spodocybe* species. *Spodocybe* and *Ampulloclitocybe* were sister clades (BP = 78%, PP = 0.99), then further clustered with *Cantharocybe* (BP = 59%, PP = 0.97) and finally together with *Cuphophyllus* formed an independent lineage (BP = 85%, PP = 1.00). Meanwhile, this lineage (Cuphophylloideae) comprising the four genera was well-supported (BP = 83%, PP = 1.00) as sister to the rest of the Hygrophoraceae.

**Figure 3.**
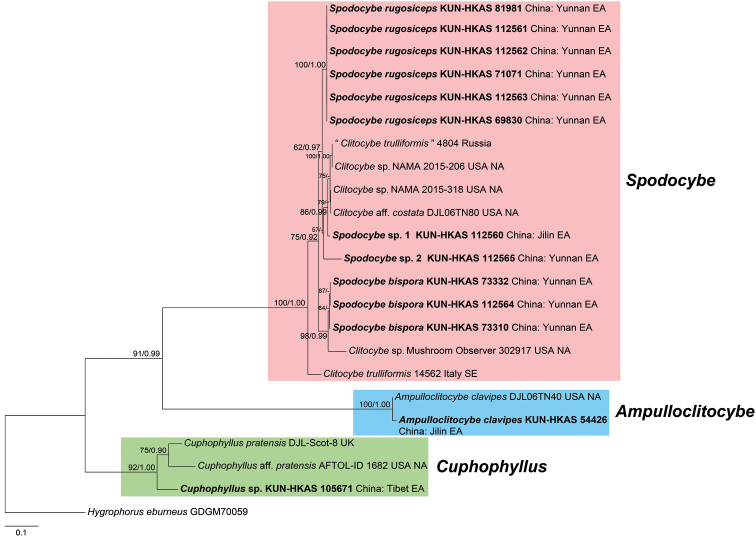
Phylogram showing the phylogenetic relationships among *Spodocybe* species and species of *Clitocybe* treated from Genbank based on ITS sequence data, with representatives of *Ampulloclitocybe*, *Cuphophyllus* and *Hygrophorus* as outgroups (rooted with *Hygrophorus eburneus*). Bootstrap values (BP) ≥ 50% from ML analysis and Bayesian posterior probabilities (PP) ≥ 0.90 are shown at nodes. The newly generated sequences are shown in bold. EA, NA and SE refer to East Asia, North America and South Europe, respectively.

In addition, an ITS dataset (23 sequences, 1053 positions) was applied to phylogenetic analysis for displaying the relationships among *Spodocybe* species from this study and species of *Clitocybe* treated from GenBank. In the ITS tree (Fig. 3), *Spodocybe* species formed a highly supported monophyletic clade with *C. trulliformis* and related species (BP = 100%, PP = 1.00), which was also a sister clade to *Ampulloclitocybe* with strong support (BP = 91%, PP = 0.99).

### Taxonomy

#### Cuphophylloideae

Taxon classificationFungiAgaricalesHygrophoraceae

Z. M. He & Zhu L. Yang subf. nov.

C96C3465-EDF8-5E8F-B6E1-C1FDD9144C40

839377

##### Diagnosis.

Characterized generally by clitocyboid basidiomes, convex to funnel-shaped pileus, decurrent lamellae, absence of veils, inamyloid basidiospores and presence of clamps.

##### Etymology.

From the type genus *Cuphophyllus*.

##### Type genus.

*Cuphophyllus* (Donk) Bon.

##### Description.

Basidiomes small, medium-sized to large, mostly clitocyboid, rarely omphalinoid or mycenoid; veils absent. Pileus convex, applanate to funnel-shaped; surface usually dry, smooth, lubricous or rarely viscid. Lamellae decurrent to deeply decurrent. Basidiospores ellipsoid, oblong or subglobose, thin-walled and inamyloid. Pileipellis usually a cutis, sometimes ixocutis or trichoderm. Lamellar trama regular, subregular, interwoven or bidirectional. Clamp connections present.

##### Habitat, ecology and distribution.

Usually gregarious or caespitose on ground, rarely on wood; widespread in temperate and tropical regions.

The genera *Ampulloclitocybe*, *Cantharocybe*, *Cuphophyllus* and *Spodocybe* are included in the subfamily Cuphophylloideae, which is in correspondence with Cuphophylloid grade of Lodge et al. (2014) plus *Spodocybe*.

#### Spodocybe

Taxon classificationFungiAgaricalesHygrophoraceae

Z. M. He & Zhu L. Yang gen. nov.

F15F630C-60D4-54EE-9717-AA0BF80B6081

839050

##### Diagnosis.

Differs from *Ampulloclitocybe* by its small basidiomes and subregular lamellar trama rather than medium-sized basidiomes and bidirectional lamellar trama. Differs from *Cuphophyllus* in the ratio of basidia to basidiospore length less than 5, and lamellar trama subregular rather than interwoven. Differs from *Cantharocybe* in its absence of cheilo- and caulocystidia, having small basidiomes rather than large ones and having subregular lamellar trama rather than regular one.

##### Etymology.

*Spodo*- refers to grey; -*cybe* refers to head; that is a *Clitocybe*-like genus with gray pileus.

##### Type species.

*Spodocybe rugosiceps* Z. M. He & Zhu L. Yang.

##### Description.

Basidiomes small, clitocyboid. Pileus convex, applanate to infundibuliform; surface dry, greyish (2B1), grey-brown (5C4) to dark grey-brown (5E4); center depressed with age. Lamellae decurrent to deeply decurrent, white (1A1) to cream (1A2), thin, moderately crowded, sometimes furcate and interveined. Stipe central, subcylindrical, concolorous with pileus. Basidiospores ellipsoid, oblong to cylindrical, colourless, hyaline, smooth, thin-walled, inamyloid; ratio of basidia to basidiospore length less than 5. Pileipellis and stipitipellis a cutis. Lamellar trama subregular. Clamp connections abundant, present in all parts of basidiome.

##### Habitat, ecology and distribution.

Saprophytic, usually gregarious or caespitose on the ground of coniferous or coniferous and broad-leaved mixed forest; distributed in the temperate and subtropical zones from June to November.

#### Spodocybe rugosiceps

Taxon classificationFungiAgaricalesHygrophoraceae

Z. M. He & Zhu L. Yang sp. nov.

CCB3B84F-91A5-5DAD-B5A5-E6CC5355CF68

839052

Figs 4A, B , 5

##### Diagnosis.

Differs from *S. bispora* in having a rugose pileus, smaller basidiospores and 4-spored rather than 2-spored basidia. Differs from *C. trulliformis* in having smaller basidiospores and a rugose rather than felty-squamulose pileus.

##### Etymology.

*rugosiceps* refers to the rugose pileus.

##### Type.

China. Yunnan Province: Kunming City, near Yeya Lake, at 25.136658°N, 102.873027°E, alt. 2000 m, 11 Aug 2020, Z. M. He 72 (KUN-HKAS 112563, holotype).

##### Description.

Basidiomes small, clitocyboid. Pileus 0.5–2 cm in diam, at first nearly applanate, then concave; surface dry and rugose, gray-brown (5E2-4) to gray-black (4F2-4) in the center and gray-brown (5C2-4) or gray (5B1-2) towards margin; center often slightly umbonate; margin straight and undulating; context thin and white (1A1) to cream (1A2). Lamellae deeply decurrent, white (1A1) to cream (1A2), thin (up to 2 mm high), crowded, sometimes forked and intervenose. Stipe 2.5–6 × 0.2–0.4 cm, central, narrowly cylindrical to subcylindrical, sometimes flexuous, hollow; surface dry and nearly smooth, concolorous with pileus; context white (1A1).

Basidiospores [60/3/3] 5–6 (6.5) × (2.5)3–3.5(4) μm, Q = (1.38)1.55–1.95(2), Qm = 1.73 ± 0.14, elongate, colorless, hyaline, smooth, thin-walled, inamyloid. Basidia 20–24 × 5–6 μm, clavate, 4-spored, colorless, hyaline, thin-walled; sterigmata up to 4 μm long; ratio of basidia to basidiospore length values about 3–5. Cystidia absent. Lamellar trama subregular; hyphae colorless, hyaline, cylindrical, thin-walled, 3–10 µm wide. Pileipellis a cutis, but in places upright or trichodermial in appearance, made up with thin-walled cylindrical hyphae 3–9 µm wide. Stipitipellis a cutis, composed of thin-walled cylindrical hyphae 3–10 μm wide. Clamp connections present in all parts of basidiome.

**Figure 4.**
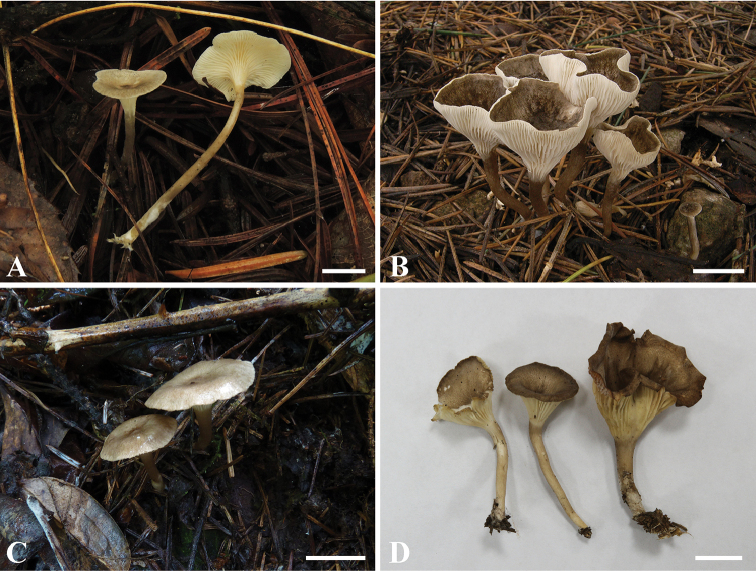
Basidiomes of described *Spodocybe* species. **A, B***Spodocybe rugosiceps* (KUN-HKAS 112563, KUN-HKAS 112562, respectively) **C, D***Spodocybe bispora* (KUN-HKAS 73332, KUN-HKAS 112562, respectively). Scale bars: 1 cm.

##### Habitat, ecology and distribution.

Gregarious or caespitose, growing saprotrophically in forest litter, often under conifers, on the ground, known from subtropical zone of Yunnan, China; from July to October.

##### Additional specimens examined.

China. Yunnan Province: Dali Bai Autonomous Prefecture, Yunlong Country, Tianchi National Nature Reserve, at 25.850365°N, 99.274236°E, alt. 2509 m, 28 Sep 2019, X. H. Wang 7471 (KUN-HKAS 112561); Kunming City, Fangwang Tree Farm, at 25.063737°N, 102.870690°E, alt. 2262 m, 22 Sep 2011, Z. L. Yang 5586 (KUN-HKAS 71071); Kunming City, Kunming Institute of Botany, at 25.147081°N, 102.748855°E, alt. 1990 m, 24 Aug 2020, Z. L. Yang 6391 (KUN-HKAS 112562); Kunming City, Qiongzhu Temple, at 25.071304°N, 102.630934°E, alt. 1900 m, 28 Jul 2013, T. Guo 779 (KUN-HKAS 81981); Yulong Country, Lashi Village, at 26.883902°N, 100.234594°E, alt. 2655 m, 31 Jul 2011, L. P. Tang 1369 (KUN-HKAS 69830).

#### Spodocybe bispora

Taxon classificationFungiAgaricalesHygrophoraceae

Z. M. He & Zhu L. Yang sp. nov.

EB4F3677-029E-51F4-A106-7B5D99A2D117

839054

Figs 4C, D , 6

##### Diagnosis.

Differs from *S. rugosiceps* in having a nearly smooth pileus, larger basidiospores and 2-spored rather than 4-spored basidia. Differs from *C. trulliformis* in having a nearly smooth rather than felty-squamulose pileus.

##### Etymology.

*Bispora* refers to 2-spored.

##### Type.

China. Yunnan Province: Baoshan City, Longyang District, Shuizhai Village, at 25.273967°N, 99.306216°E, alt. 2400 m, 12 Aug 2011, J. Qin 324 (KUN-HKAS 73310, holotype).

##### Description.

Basidiomes small, clitocyboid. Pileus 1.5–3 cm in diam, plano-convex to funnel-shaped; surface dry and nearly smooth, greyish-brown (4B2-3) to grey-brown (4E3-5); center depressed, usually with a low umbo, somewhat darker; margin generally straight and undulating, incurved when old; context thin and white (1A1). Lamellae deeply decurrent, white (1A1) to cream (1A2), thin, 1–2 mm high, relatively crowded, sometimes forked and intervenose. Stipe 1–3 × 0.2–0.4 cm, central, subcylindrical, hollow; surface dry and nearly smooth, concolorous with pileus; context white (1A1).

**Figure 5.**
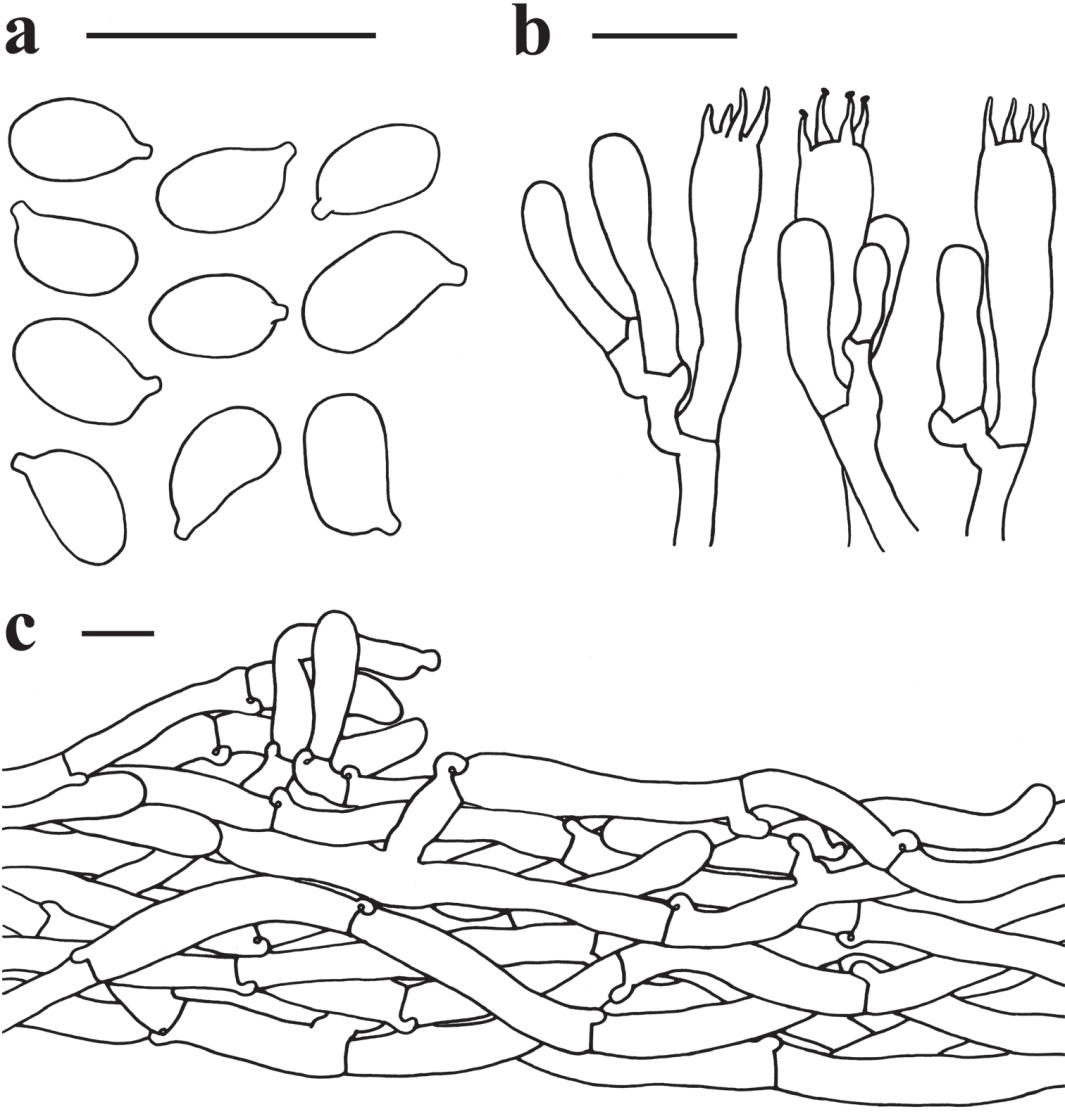
Microscopic features of *Spodocybe rugosiceps* (KUN-HKAS 112563, holotype) **a** basidiospores **b** basidia **c** pileipellis. Scale bars: 10 μm.

Basidiospores [60/3/3] (7)7.5–10.5(11.5) × 3–4 μm, Q = (2.05)2.11–3(3.33), Qm = 2.56 ± 0.3, cylindrical, colorless, hyaline, smooth, thin-walled, inamyloid. Basidia 20–30 × 4–5.5 μm, clavate, 2-spored, colorless, hyaline, thin-walled; sterigmata up to 10 μm long; ratio of basidia to basidiospore length less than 5 (about 2–4). Cystidia absent. Lamellar trama subregular, colorless, hyaline, made up of thin-walled cylindrical hyphae with 3–10 µm wide. Pileipellis a cutis, composed of thin-walled cylindrical hyphae 3–11 µm wide. Stipitipellis a cutis, composed of thin-walled cylindrical hyphae 3–10 μm wide. Clamp connections in all parts of basidiomes.

##### Habitat, ecology and distribution.

Saprophytic, usually gregarious on the ground of coniferous or coniferous and broad-leaved mixed forest, known from Yunnan, China; July to September.

##### Additional specimens examined.

China. Yunnan Province: Kunming City, Qipan Mountain, at 26.060020°N, 102.576823°E, alt. 1900 m, 25 Jul 2020, Z. M. He 35 (KUN-HKAS 112564); Nujiang City, Lanping Country, No. 311 Provincial Highway, at 26.636613°N, 99.557809°E, alt. 2660 m, 14 Aug 2011, J. Qin 346 (KUN-HKAS 73332).

**Figure 6.**
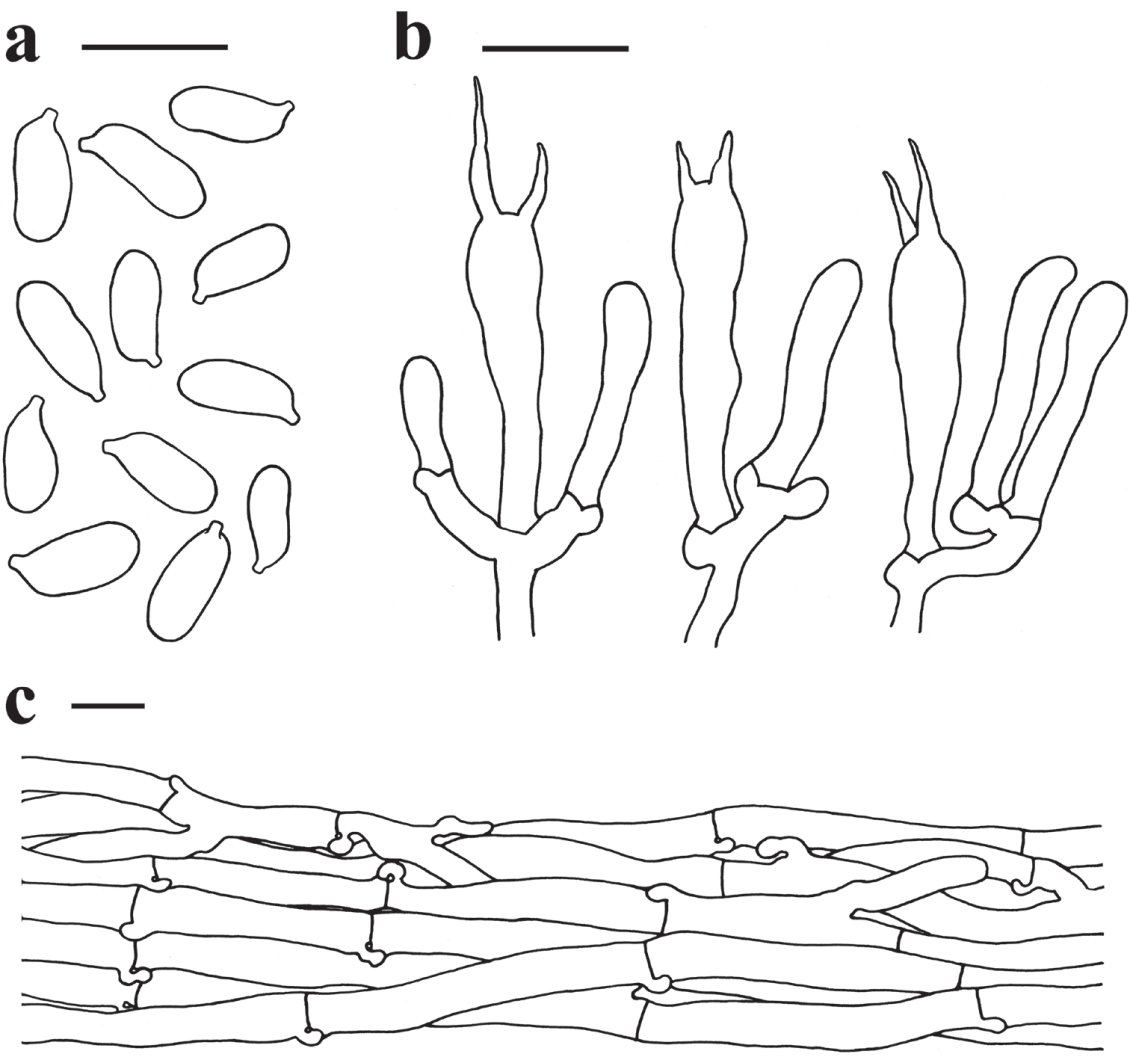
Microscopic features of *Spodocybe bispora* (KUN-HKAS 73310, holotype) **a** basidiospores **b** basidia **c** pileipellis. Scale bars: 10 μm.

## Discussion

### The new genus *Spodocybe*

In our current study, the new clitocyboid species were clustered into a monophyletic lineage (BP = 100%, PP = 1.00) in the Hygrophoraceae according to the multi-gene phylogenetic analysis (Figs 1, 2). As a result, the new generic name *Spodocybe* is proposed here to accommodate the new lineage, which is irrelevant to Clitocybeae of the Tricholomatoid clade (Matheny et al. 2006; Alvarado et al. 2015). The three-gene tree of the Hygrophoraceae (Fig. 1) in this study presented basically consistent topological structure with Lodge et al. (2014), and showed that *Spodocybe* was a sister to *Ampulloclitocybe* located within the family Hygrophoraceae and further confirmed by a six-gene tree (Fig. 2).

Besides the molecular analyses, morphological data also support its separation from the relative genera. *Spodocybe* shares clitocyboid basidiomes, decurrent lamellae, inamyloid basidiospores and the presence of clamps with the other genera *Ampulloclitocybe*, *Cuphophyllus* and *Cantharocybe*. However, the genus *Ampulloclitocybe*, typified by *A. clavipes*, differs from *Spodocybe* in having medium-sized basidiomes and bidirectional lamellar trama (Harmaja 2002; Lodge et al. 2014). Afterwards, *Cuphophyllus* differs from *Spodocybe* in having long basidia, typically 7−8 (rarely 5−6) times the length of the basidiospores, highly interwoven lamellar trama, rarely subregular (Voitk et al. 2020). Finally, *Cantharocybe* differs from *Spodocybe* in having large basidiomes, broad lamellae, cheilo- and caulocystidia, clamps but not on all hyphal septa or at the base of every basidium and more regular lamellar trama (Ovrebo 2011; Hosen et al. 2016). In view of the four genera above with different structures in lamellar trama (Fig. 2), the type of lamellar trama can become a good distinguishing microscopic character for them.

For a long time, *C. trulliformis* has been placed in the genus *Clitocybe* based on the clitocyboid feature and habit since 1879 (Karsten 1879). However, *C. trulliformis* shares many morphological characteristics with *Spodocybe*, such as the small basidioma with applanate to infundibuliform pileus, grey-brown pileus and stipe, decurrent and whitish lamellae, and smooth and inamyloid basidiospores (Bas et al. 1995). Besides, the ITS phylogenetic analysis in our study (Fig. 3) showed that *C. trulliformis* and related *Clitocybe* species were involved in the *Spodocybe* clade as well, indicating that *C. trulliformis* and related species should be placed with *Spodocybe*. In consequence, it is foreseeable that *C. trulliformis* and other related clitocyboid species will eventually be moved to *Spodocybe*. Accordingly, more taxonomic work is needed in future.

### The placements of *Spodocybe*, *Cuphophyllus*, *Ampulloclitocybe* and *Cantharocybe*

In previous studies, *Cuphophyllus*, *Ampulloclitocybe* and *Cantharocybe* were treated as basal in Hygrophoraceae (Lodge et al. 2014), but their phylogenetic placements were not resolved. In a six-gene phylogenetic analysis by Binder et al. (2010) and a three-gene analysis by Wang et al. (2018), *Ampulloclitocybe* and *Cantharocybe* were located between *Cuphophyllus* and the rest of the Hygrophoraceae, but without support. While two four-gene analyses by Lodge et al. (2014) showed that *Ampulloclitocybe* and *Cantharocybe* were sister clades as basal to *Cuphophyllus* along with the rest of the Hygrophoraceae with weak support. However, in our six-gene analysis (Fig. 2), the new proposed genus *Spodocybe* and *Ampulloclitocybe* were sisters (BP = 78%, PP = 0.99) and they clustered with *Cantharocybe* followed by *Cuphophyllus*, forming a supported monophyletic sister clade to the rest of the Hygrophoraceae (BP = 83%, PP = 1.00). Hence, *Spodocybe*, *Ampulloclitocybe*, *Cantharocybe* and *Cuphophyllus* should be retained in Hygrophoraceae, and a new subfamily, Cuphophylloideae, is proposed to accommodate the lineage.

## Supplementary Material

XML Treatment for Cuphophylloideae

XML Treatment for Spodocybe

XML Treatment for Spodocybe rugosiceps

XML Treatment for Spodocybe bispora

## Supplementary materials

Supplementary material 1 Alignment of ITS-LSU-RPB2 dataset used in the three-gene phylogenetic analysis Data type fasta file Explanation note ITS: 1-1380, LSU: 1381–2356, RPB2: 2357–3135. https://binary.pensoft.net/file/536280This dataset is made available under the Open Database License (http://opendatacommons.org/licenses/odbl/1.0/). The Open Database License (ODbL) is a license agreement intended to allow users to freely share, modify, and use this Dataset while maintaining this same freedom for others, provided that the original source and author(s) are credited.Zheng-Mi He, Zhu L. Yang

Supplementary material 2 Alignment of ITS-LSU-RPB1-RPB2-TEF1-ATP6 dataset used in the six-gene phylogenetic analysis Data type fasta file Explanation note ITS: 1–1217, LSU: 1218–2158, RPB1: 2159–3358, RPB2: 3359–4089, TEF1: 4090–4967, ATP6: 4968–5405 https://binary.pensoft.net/file/536281This dataset is made available under the Open Database License (http://opendatacommons.org/licenses/odbl/1.0/). The Open Database License (ODbL) is a license agreement intended to allow users to freely share, modify, and use this Dataset while maintaining this same freedom for others, provided that the original source and author(s) are credited.Zheng-Mi He, Zhu L. Yang

Supplementary material 3 Alignment of ITS dataset used in the single-gene phylogenetic analysis Data type fasta file https://binary.pensoft.net/file/536282This dataset is made available under the Open Database License (http://opendatacommons.org/licenses/odbl/1.0/). The Open Database License (ODbL) is a license agreement intended to allow users to freely share, modify, and use this Dataset while maintaining this same freedom for others, provided that the original source and author(s) are credited.Zheng-Mi He, Zhu L. Yang
